# Reproductive toxicity of sodium valproate in male rats

**DOI:** 10.4103/0253-7613.64503

**Published:** 2010-04

**Authors:** Laxminarayana Bairy, Vijay Paul, Yeshwanth Rao

**Affiliations:** Department of Pharmacology, Kasturba Medical College, Manipal, India; 1 Department of Anatomy, Maleka Manipal Medical College, Manipal, India; 2 Department of Pharmacology, Maleka Manipal Medical College, Manipal, India

**Keywords:** Cytoarchitecture of testis, sodium valproate, sperm count, sperm morphology, sperm motility

## Abstract

**Objectives:**

To assess the effects of sodium valproate on rat sperm morphology, sperm count, motility, and histopathological changes in testis.

**Materials and Methods:**

Male Wistar rats (12 week old) were treated with sodium valpraote and sacrificed at the end of 2^nd^, 4^th^, 5^th^, 7^th^, 10^th^ and 15^th^ week after the last exposure to sodium valproate. Epididymal sperm count, sperm motility, sperm morphology, and histopathology of testes were analyzed.

**Results:**

Sperm count and sperm motility were decreased significantly by sodium valproate. The percentage of abnormal sperms increased in a dose-dependent manner. A histopathological study revealed that sodium valproate had caused sloughing of epithelial cells in testes.

**Conclusion:**

Sodium valproate causes reversible change in sperm motility, sperm count, morphology, and cytoarchitecture of testes.

## Introduction

Epilepsy is a curable disease, which necessitates continuous treatment with optimal dose of antiepileptic drugs. Sodium valproate is one of the drugs of choice in primary generalized tonic-clonic seizures,[1] absence seizures, and myoclonic seizures.[2] It is also the drug of choice in epileptic syndromes such as the Lennox-Gastaut syndrome because of its wide therapeutic spectrum, and it may be useful in infantile spasms.[3,4] Oral administration for 60 days in rats significantly decreased testicular weight, sperm cell concentration, live sperms, and percentage of progressively motile spermatozoa and increased percentage of morphologically abnormal spermatozoa.[5] It has been reported that in epileptic children receiving valproic acid, the latter produces a significant change in sister chromatid exchange.[6] It is also reported that it lacks mutagenic potential within the therapeutic dose range when administered chronically to adult male patients with epilepsy.[7]

There is a report of dose-dependent effect of chronic valproate treatment on testicular morphology in rats.[8] Nishimura *et al*[9] suggest that sperm motility and histopathological evaluation of testes are sensitive methods for assessing toxicity of valproate on male reproductive organs. However, there is a paucity of reports on the effects of valproate on reproductive toxicity with regard to its time, duration, and reversibility. Hence, a study was planned to assess the effects of sodium valproate on sperm morphology, sperm count, motility, and histopathological changes in epididymis and testis.

## Materials and Methods

### Animals

Twelve week old male Wistar rats (150-200g) bred locally in the central animal house were selected for the study. They were housed in propylene cages and were provided bedding with paddy husk. Temperature was maintained at 25 ± 1°C with a humidity of 45 ±1%. Animals had free access to sterile food (animal chow) and water *ad libitum*. Animal care and handling was done as per the guidelines set by the Indian National Academy New Delhi, India. The study was started after getting clearance from the institutional animal ethics committee. The registration number of animal facility is 94/1999/CPCSEA.

A total of 144 rats were segregated to 24 groups of 6 animals each. Six groups each were treated with 0.1 ml of distilled water, gum acacia control, sodium valproate 200 mg, and sodium valproate 400 mg for 60 days (N=6/group/dose/sample time). Rats were sacrificed by terminal anesthesia (pentobarbital sodium, 45 mg/kg) at the end of 2^nd^, 4^th^, 5^th^, 7^th^, 10^th^, and 15^th^ week after the last exposure to sodium valproate. The sacrifice time points represent the sampling of spermatozoa in the epididymis and testis, spermatids, primary spermatocytes, secondary spermatocytes, spermatogonia, and stem cells (10^th^ and 15^th^ week), respectively.[10‐12]

### Chemicals

The powdered form of sodium valproate was obtained from Knoll Pharmaceuticals Ltd, Mumbai. The median lethal dose of sodium valproate in rodents varies between 1100 and 3900 mg/kg body weight.[13] The dose and route of administration were selected based on earlier reports.[8,14] The powdered form of sodium valproate was weighed using an electronic weighing balance and was dissolved in water and administered orally.

### Epididymal Sperm Count and Sperm Motility

The epididymal sperm suspension is prepared in 1 ml of phosphate buffered saline (PBS) at pH 7.2. The sperm count was determined in a hemocytometer. An aliquot from the suspension (1 ml) was diluted 1:40 with PBS. A sample of the diluted suspension is charged into a counting chamber (Neubauer's chamber). The total sperm count in eight squares (Except the central erythrocyte area) of 1 mm^2^ each was determined and multiplied by 5 × 10^4^ to get the total count. Sperm motility was also counted in same eight squares and percentage of motile sperms was recorded.

### Sperm Morphology Assay

A fine suspension was made and stained with 0.2 ml of 1% aqueous eosin. About one drop of stained suspension was placed on the clean slide. It was dried, cleaned, and mounted in Di-N-Butyle Phthalate in Xylene (DPX). Slides were looked for sperm-shape abnormality, 1000 sperms/animal being scored. Sperms were classified into normal and abnormal sperms. The abnormal sperms were classified under head abnormalities and tail abnormalities. The head abnormalities were classified as amorphous, hook less, banana shaped, double headed, and bent. The tail abnormalities were classified as coiled/folded and double tailed.

### Histopathology of Testis

The testes/epididymis were removed and fixed in Bouin's fluid for 24 h. After excessive washing in 70% alcohol, the tissue was processed for paraffin embedding and 5 *μ* thick paraffin sections were stained with hematoxylin and eosin. The sections were analyzed for the presence or absence of vacuoles, gaps, and abnormal cells.

### Seminiferous Tubular Diameter (STD) and Epithelial Height (SEH)

The diameters of 20 transversely cut tubules were measured using ocular micrometer calibrated with the stage micrometer (Erna Opticals, Japan). In each tubule, two measurements were made one perpendicular to the other and their average is taken. The epithelial height was measured in ten tubules for each animal. In each tubule, the SEH was measured from the basement membrane to the surface of the epithelium at two different regions and the mean was taken.

### Statistical Analysis

Six animals were used in each group and mean ± SD (standard deviation) was calculated. Results were analyzed by one-way Analysis of Variance (ANOVA). Values of *P*< 0.05 were considered statistically significant.

## Results

### Effect on Sperm Count

**Figure 1:**
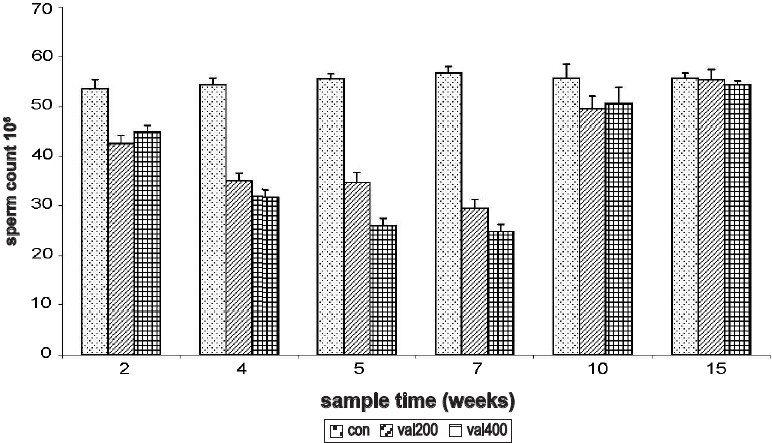
Time response relationship for sodium valproate-induced changes in sperm count. Con = control, val 200 = Sodium valproate 200 mg/kg, val 400 = Sodium valproate 400 mg/kg. Each time at particular dose represent mean +SD from six animals. Significant values are: normal control vs. treated, 1= *P*< 0.05, 3 = *P*<0.001; 200 mg vs. 400 mg, a = *P*<0.05, b = *P*<0.01, c = *P*<0.001.

Both 200 mg/kg and 400 mg/kg of sodium valproate significantly decreased sperm count, starting from the 2^nd^ week sampling time and continued through the 4^th^, 5^th^, and 7^th^ week. The lowest sperm count was observed during the 7^th^ week and the sperm count returned to control levels by 15^th^ week. Complete recovery was observed by 15^th^ week and the recovery was almost the same in both the doses [Figure 1].

### Effect on Sperm Motility

**Figure 2:**
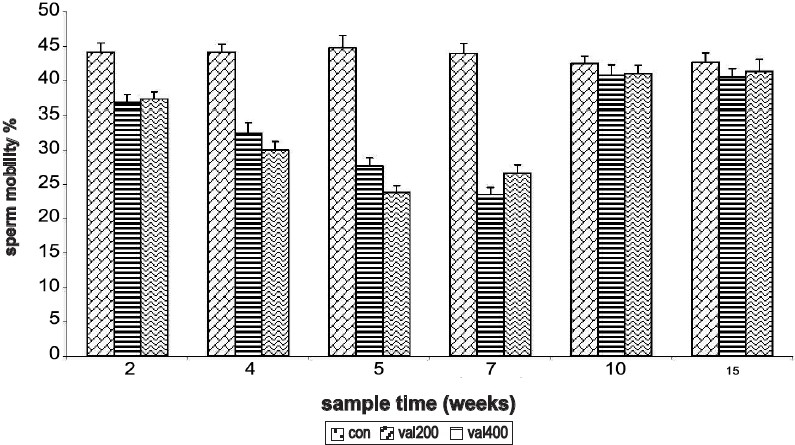
Time response relationship for sodium valproate-induced changes in sperm motility. Con = control, val 200 = Sodium valproate 200 mg/kg, val 400 = Sodium valproate 400 mg/kg. Each time at particular dose represent mean +SD from six animals. Significant values are: normal control vs. treated, 3 = *P*<0.001; 200 mg vs. 400mg, a = *P*<0.05, b = *P*<0.01.

Sperm motility was significantly decreased significantly at 2^nd^, 4^th^, 5^th^, and 7^th^ week with both doses of the drug. By the 10th week, there was complete recovery and the sperm motility values reached the control values. For the rats treated with 200 mg/kg sperm motility was least during the 7^th^ week sampling time and for 400 mg/kg, sperm motility was least at 5^th^ week sampling time. Recovery period to normal values of sperm motility was same in both 200 mg/kg and 400 mg/kg treated rats and reached complete recovery by the 10^th^ week sampling time [Figure 2].

### Effect on Sperm Morphology

**Figure 3:**
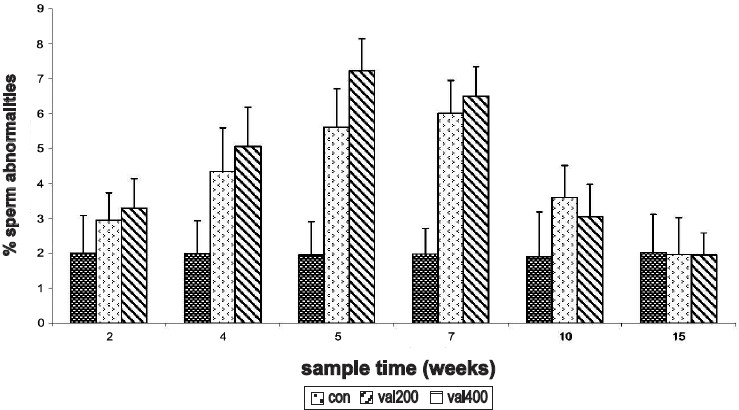
Time response relationship for sodium valproate-induced changes in sperm morphlogy. Con = control, val 200 = Sodium valproate 200 mg/kg, val 400 = Sodium valproate 400 mg/kg. Each time at particular dose represent mean +SD from six animals. Significant values are: normal control vs. treated, 1= *P*< 0.05, 2 = *P*<0.01, 3 = *P*<0.001

The percentage of abnormal sperms increased significantly in a time-dependent manner at 4^th^, 5^th^, and 7^th^ week sampling time in rats treated with both the doses. The maximum sperm abnormality was observed at the 5^th^ week with the higher dose [Figure 3] and during the 7^th^ week sampling time in rats treated with 200 mg/kg.

### Effect on the Microscopic Architecture of Testis

**Figure 4:**
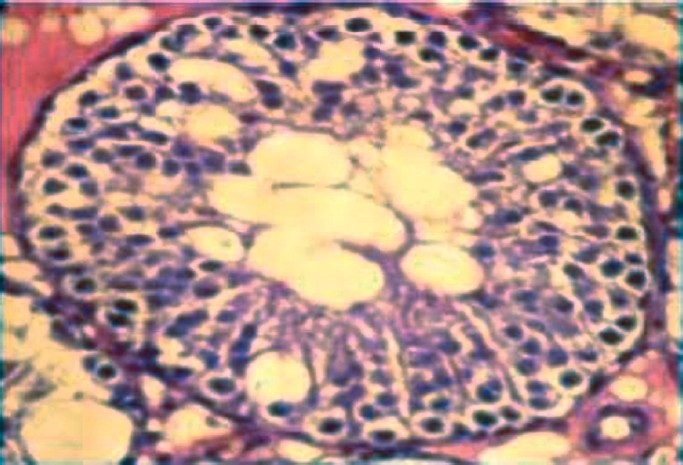
Cross section of testes of rats treated with 400 mg sodium valproate for 7 weeks showing the presence of vacuoles. Magnification: 400× (Scale bar:=25μ).

**Figure 5:**
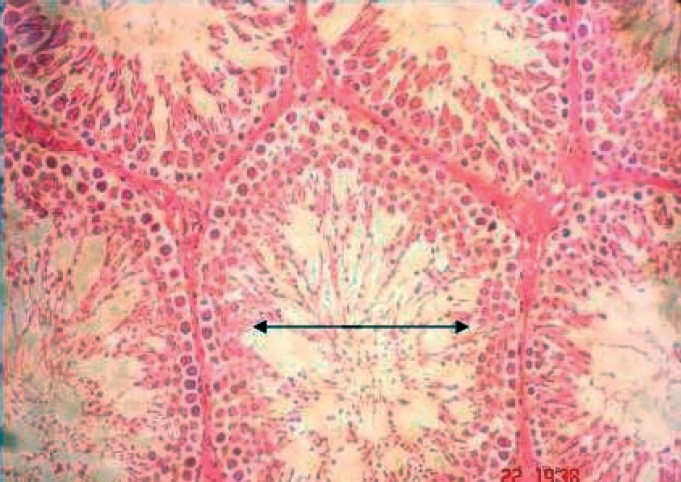
Cross section of the testes with the arrow indicating the increased diameter in the lumen of the seminiferous tubule.Magnification: 400× (Scale bar=25μ).

Sloughing of epithelial cells in testes was observed in the rats treated with lower as well as with higher dose of the drug. The sloughed cells were found in the lumen of the seminiferous tubules. The presence of vacuoles was seen at both the doses. However, the maximum number of vacuoles appeared at 5^th^ and 7^th^ week with the higher dose. Atrophy of the tubules was rarely seen and multinucleated cells were absent [Figures 4 and 5].

### Effect on the Gross Architecture of Testis

**Figure 6:**
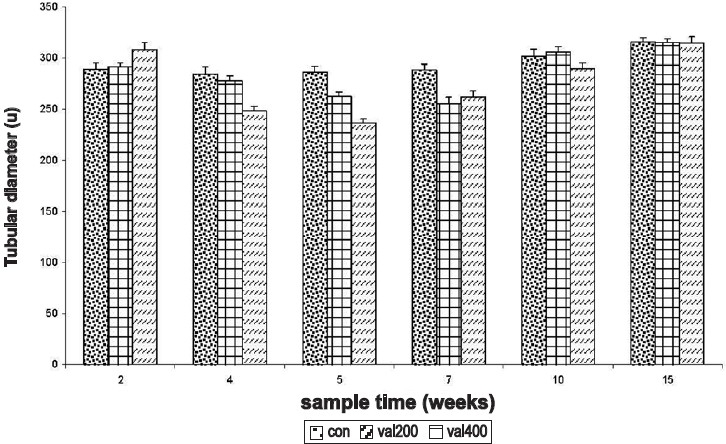
Time response relationship for sodium valproate-induced changes in seminiferous tubular diameter. Con = control, val 200 = Sodium valproate 200 mg/kg, val 400 = Soduim valproate 400 mg/kg.Each time at particular dose represent mean +SD from six animals.Significant values are: normal control vs. treated, 1= *P*< 0.05, 3 = *P*<0.001; 200 mg vs. 400 mg, b = *P*<0.01, c = *P*<0.001.

Sodium valproate did not alter the weight of the testes significantly. However, the diameter of the seminiferous tubule was significantly decreased in the rats treated with the higher dose in 4^th^, 5^th^, and 7^th^ week samples. However, the tubular diameter was significantly increased in rats treated with the higher dose at 2^nd^ week sampling time. Complete recovery of the tubular diameter was seen only at the 15^th^ week [Figure 6].

**Figure 7:**
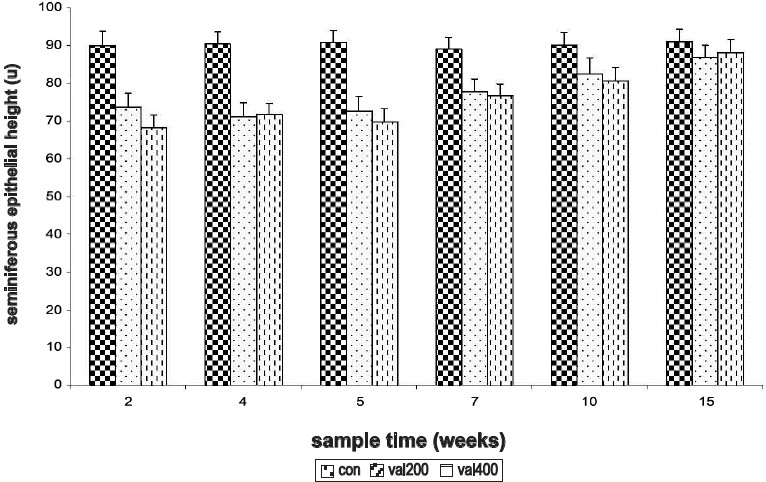
Time response relationship for sodium valproate-induced changes in seminiferous epithelial height. Con = control, val 200 = Sodium valproate 200 mg/kg, val 400 = Sodium valproate 400 mg/kg. Each time at particular dose represent mean +SD from six animals. Significant values are: control vs. treated 1 = *P*<0.05, 2 = *P*<0.01, 3 = *P*<0.001.

The epithelial height of the tubules was significantly reduced at all sampling weeks except for the 15^th^ week, regardless of the dose. Maximum reduction of the epithelial height was observed at 5^th^ week in both doses of the drug. Recovery period for both the doses was long and normal height was attained only by the 15^th^ week [Figure 7].

## Discussion

Sperm count is one of the most sensitive tests for spermatogenesis, since it gives the cumulative result of all stages in sperm production, and it is highly correlated with fertility.[15] Our results show that sodium valproate is cytotoxic to the sperm since it decreases the sperm count significantly in a linear manner from 2^nd^ to 7^th^ week sampling time, regardless of the dose. The duration of spermatogenic cycle in rats is 52 to 60 days and our findings point out that the germ cells affected are approximately the spermatids, spermatocytes, and spermatogonia. The count was reduced even at the end of the 10^th^ week. This in all probability signifies its effect on the stem cells. The decline in sperm count was highest at the 7^th^ week, which possibly indicates that the spermatogonia are more vulnerable to the toxic effects of sodium valproate.

Since the very earliest (spermatogonial) phase is when nearly all cell multiplication occurs, chemicals which interfere with this phase will probably have a disproportionately greater effect on sperm output than chemicals acting during the spermatid phase.[16] It was not mutagenic to the sperms at 7^th^ week. This can be demonstrated by the reduced number of abnormal sperms observed by the end of that week.

A study conducted to determine the gonadotoxic effect of sodium valproate found that serum testosterone levels were not significantly changed, but there was a highly significant increase in FSH and LH concentrations at the high dose.[14] However, an earlier study by Soliman *et al*[5] had reported a decrease in plasma testosterone, FSH and LH and an increase in prolactin levels on treatment with sodium valproate. The decrease in the sperm count in the present study may be due to the decreased levels of intratesticular testosterone at the 2^nd^ to 7^th^ week sampling time, as testosterone level is directly linked to spermatogenesis.[17] It is also possible that the Sertoli cells might have been affected and the other possibility might be due its effect on the epididymal function. According to Ameen *et al*[18] recovery of sperm count was complete at the low dose level (270 mg/kg), but was incomplete at the high dose level (540 mg/kg), 8 weeks after discontinuing valproate. In contrast, in the present study regardless of the dose, there was a reduction in the sperm count 7 weeks after discontinuing the drug. However, reversal of the harmful effects on the sperms was observed by the end of the 15^th^ week, which indicates that the stem cells were not affected severely.

Studies have examined rat sperm motility as a reproductive end-point[19‐22] and sperm motility assessments are an integral part of some reproductive toxicity test guidelines.[19,20,23‐25] Sodium valproate decreased the percentage of sperm motility in a time-dependent manner. Similar results were observed by earlier workers.[11,26] The sperm motility largely depends on the microtubular apparatus of the sperm tail.[26] In the current study it was also observed that a considerable number of abnormal sperms were with a defect in their tail. Valproate is known to compromise mitochondrial function.[27] Mitochondria are needed for the energy production of the cells and motility of the sperm requires normal mitochondrial function and therefore mitochondrial effects of the valproate might be a cause of reduced sperm motility.[28]

According to Russel and Russel,[29] male germ cells are very ideal and easy for the study of the genotoxicity of drugs since they exist in different phases of cell development and differentiation. Genotoxic effects of the drug would result in morphologically abnormal sperms and therefore the counting and classification of the types of abnormal sperm can determine the presence and extent of genotoxicity.[30] Sodium valproate treatments resulted in more than double the percentage of abnormal sperms and hence it could be considered as mutagen. Currently it is widely accepted that the induction of sperm abnormality mainly takes place through point mutation.[31,32] Therefore, it is possible that change in the sperm structure might have been due to point mutation.

The question as to why the higher dose of sodium valproate increased the tubular diameter is a little difficult to address. The most likely reason would be the extensive sloughing that was seen at 2^nd^ week sampling time. According to Nakai *et al*,[33] the sloughed cells can block the efferent ductules and hence increase the diameter of the tubules.

## Conclusion

This study concludes that sodium valproate has a reversible mutagenic effect on the germ cells and somatic cells. Findings from this study point out the gonadotoxic and cytotoxic potential of this drug.

## References

[1] Semah F, Picot MC, Derambure P, Dupont S, Vercueil L, Chassagnon S (2004). The choice of antiepileptic drugs in newly diagnosed epilepsy: A national French survey. Epileptic Disord.

[2] Mattson RH, Cramer JA, Collins JF, Smith DB, Delgado-Escueta AV, Browne TR (1985). Comparison of carbamazepine, phenobarbital, phenytoin, and primidone in partial and secondarily generalized tonic-clonic seizures. N Engl J Med.

[3] Oka E, Murakami N, Ogino T, Kobayashi K, Ohmori I, Akiyama T (2004). Initiation of treatment and selection of antiepileptic drugs in childhood epilepsy. Epilepsia.

[4] Verity CM, Hosking G, Easter DJ (1995). The Paediatric EPITEG Collaborative. A multicentre comparative trial of sodium valproate and carbamazepine in paediatric epilepsy. Dev Med Child Neurol.

[5] Soliman GA, Abla Abd, el-Meguid (1999). Effects of antiepileptic drugs carbamazepine and sodium valproate on fertility of male rats. Dtsch Tierarztl Wochenschr.

[6] Hu LJ, Lu XF, Lu BQ, Huang YQ (1990). The effect of valproic acid on SCE and chromosome aberrations in epileptic children. Mutat Res.

[7] Schaumann BA, Winge VB, Garry VF (1989). Sister chromatid exchanges in adult epilepsy patients on valproate monotherapy. Epilepsy Res.

[8] Sveberg Roste L, Tauboll E, Berner A, Berg KA, Aleksandersen M, Gjerstad L (2001). Morphological changes in the testis after long-term valproate treatment in male Wistar rats. Seizure.

[9] Nishimura T, Sakai M, Yonezawa H (2000). Effects of valproic acid on fertility and reproductive organs in male rats. J Toxicol Sci.

[10] Goldberg RB, Geremia R, Bruce WR (1977). Histone synthesis and replacement during spermatogenesis in the mouse. Differentiation.

[11] Oakberg EF (1956). Duration of spermatogenesis in the mouse and timing of stages of the cycle of the seminiferous epithelium. Am J Anat.

[12] Wyrobek AJ, Bruce WR (1975). Chemical induction of sperm abnormalities in mice. Proc Natl Acad Sci U S A.

[13] Walker RM, Smith GS, Barsoum NJ, Macallum GE (1990). Preclinical toxicology of the anticonvulsant calcium valproate. Toxicology.

[14] Sveberg Roste L, Tauboll E, Isojärvi JI, Pakarinen AJ, Huhtaniemi IT, Knip M (2002). Effects of chronic valproate treatment on reproductive endocrine hormones in female and male Wistar rats. Reprod Toxicol.

[15] Meistrich ML, Finch M, da Cunha MF, Hacker U, Au WW (1982). Damaging effects of fourteen chemotherapeutic drugs on mouse testis cells. Cancer Res.

[16] Sharpe RM, Knobil E, Neill JD (1994). Regulation of spermatogenesis. The physiology of reproduction.

[17] Zirkin BR, Santulli R, Awoniyi CA, Ewing LL (1989). Maintenance of advanced spermatogenic cells in the adult rat testis: Quantitative relationship to testosterone concentration within the testis. Endocrinology.

[18] Ameen A, Erian E (1999). Toxicological effect of valproate sodium on gonadal activity in male albino rats. J Egypt Ger Soc Zool.

[19] Morrissey RE, Lamb JC, Schwetz BA, Teague JL, Morris RW (1988). Association of sperm, vaginal cytology, and reproductive organ weight data with results of continuous breeding reproduction studies in Swiss (CD-1) mice. Fundam Appl Toxicol.

[20] Morrissey RE, Schwetz BA, Lamb JC, Ross MD, Teague JL, Morris RW (1988). Evaluation of rodent sperm, vaginal cytology, and reproductive organ weight data from National Toxicology Program 13-week studies. Fundam Appl Toxicol.

[21] Toth GP, Stober JA, George EL, Read EJ, Smith MK (1991). Sources of variation in the computer-assisted motion analysis of rat epididymal sperm. Reprod Toxicol.

[22] Toth GP, Stober JA, Zenick H, Read EJ, Christ SA, Smith MK (1991). Correlation of sperm motion parameters with fertility in rats treated subchronically with epichlorohydrin. J Androl.

[23] Gray LE, Ostby J, Sigmon R, Ferrell J, Rehnberg G, Linder R (1988). The development of a protocol to assess reproductive effects of toxicants in the rat. Reprod Toxicol.

[24] Morrissey RE, Lamb JC, Morris RW, Chapin RE, Gulati DK, Heindel JJ (1989). Results and evaluations of 48 continuous breeding reproduction studies conducted in mice. Fundam Appl Toxicol.

[25] US EPA Health effects test guidelines OPPTS 870.3800: Reproduction and fertility. Washington, DC, US Environmental Protection Agency 1998a.

[26] Eddy EM, O'Brien DA, Knobil E, Neill JD (1994). The Physiology of Reproduction.

[27] Ponchaut S, Veitch K (1993). Valproate and mitochondria. Biochem Pharmacol.

[28] Isojärvi JI, Löfgren E, Juntunen KS, Pakarinen AJ, Päivänsalo M, Rautakorpi I (2004). Effect of epilepsy and antiepileptic drugs on male reproductive health. Neurology.

[29] Russell LD, Russell JA (1991). Short-term morphological response of the rat testis to administration of five chemotherapeutic agents. Am J Anat.

[30] Sharma RK, Roberts GT, Johnson FM, Malling HV (1979). Translocation and sperm abnormality assays in mouse spermatogonia treated with procarbazine. Mutat Res.

[31] Aubele M, Jütting U, Rodenacker K, Gais P, Burger G, Hacker-Klom U (1990). Quantitative evaluation of radiation-induced changes in sperm morphology and chromatin distribution. Cytometry.

[32] Chauhan LK, Pant N, Gupta SK, Srivastava SP (2000). Induction of chromosome aberrations, micronucleus formation and sperm abnormalities in mouse following carbofuran exposure. Mutat Res.

[33] Nakai M, Hess RA, Moore BJ, Guttroff RF, Strader LF, Linder RE (1992). Acute and long-term effects of a single dose of the fungicide carbendazim (methyl 2-benzimidazole carbamate) on the male reproductive system in the rat. J Androl.

